# High frequency jet ventilation at the distal end of tracheostenosis during flexible bronchoscopic resection of large intratracheal tumor: Case series

**DOI:** 10.1097/MD.0000000000019929

**Published:** 2020-06-19

**Authors:** Guang-Qiu Zhu, Xiao-Mai Wu, Dong-Hang Cao

**Affiliations:** a Department of Anesthesiology; b Department of Respiratory Medicine, Taizhou Hospital of Zhejiang Province, Wenzhou Medical University, Linhai, China.

**Keywords:** anesthesia, flexible bronchoscopy, high jet frequency ventilation, intratracheal tumor, resection, suction catheter

## Abstract

**Introduction:**

Resection of a large intratracheal tumor with severe obstruction via flexible bronchoscope remains a formidable challenge to anesthesiologists. Many artificial airways positioned proximal to tracheal obstruction can not ensure adequate oxygen supply. How to ensure effective gas exchange is crucial to the anesthetic management.

**Patient concerns:**

Five patients of intratracheal tumor occupying 70% to 85% of the tracheal lumen were scheduled for tumor resection via flexible bronchoscope.

**Diagnosis:**

The patients were diagnosed with intratracheal tumor based on their symptoms, radiographic findings and tracheoscopy.

**Interventions:**

We describe a technique of high frequency jet ventilation (HFJV) using an endobronchial suction catheter distal to tracheostenosis during the surgery, which ensured the good supply of oxygen. We applied general anesthesia with preserved spontaneous breathing. A comprehensive anesthesia protocol that emphasizes bilateral superior laryngeal nerve (SLN) block and sufficient topical anesthesia. An endobronchial suction catheter was introduced transnasally into the trachea and then advanced through the tracheostenosis with the tip proximal to the carina under direct vision with the aid of fiber bronchoscope. HFJV was then performed through the suction catheter.

**Outcomes:**

The S_P_O_2_ maintained above 97% during the surgery. Carbon dioxide retention was alleviated obviously when adequate patency of the trachea lumen achieved about 30 min after the beginning of surgery. HFJV was ceased and all patients had satisfactory spontaneous breathing at the end of the procedure.

**Conclusion:**

HFJV at the distal end of tracheostenosis is a suitable ventilation strategy during flexible bronchoscopic resection of a large intratracheal tumor.

## 1 Introduction

Interventional pulmonology is a rapidly growing subspecialty with remarkable advancements in diagnostic and therapeutic innovations in recent years.^[1–3]^ It is a great challenge for anesthesiologists to manage interventional bronchoscopy in patients with intratracheal tumor as the compromised airway is shared with surgeons.^[3,4]^ American Society of Anesthesiologists (ASA) physical status scores of the patients were the most commonly graded III or IV because of comorbidity and central airway obstruction.^[5]^ How to prevent the seepage of blood and small chunks of tumor tissue from flowing distally into the tracheobronchial tree during resection should also be considered.^[6]^

A larger size of endotracheal tube (ETT) (i.e., ID 8–9 mm) and laryngeal mask airway (LMA) (i.e., size 4 i-gel LMA) is commonly used for bronchoscopy.^[3,7,8]^ An LMA produces less stimulation at placement compared with an ETT and is a relatively nontraumatic method when a tracheal lesion is present.^[9,10]^ The LMA also allows proper evaluation and treatment of subglottic lesions.^[9]^ Thus, LMA is a common option in the context of flexible bronchoscopy, improving oxygen saturation and facilitating insertion of the bronchoscope into the larynx.^[11,12]^ If the obstruction is severe, the airway resistance is very high accompanied with air leak, and ventilation is unsatisfactory.^[13,14]^

How to manage the severe tracheal obstruction during resection via flexible bronchoscope remains uncertain. Here, we report on our experience with 5 cases. High frequency jet ventilation (HFJV) is an alternative for interventional bronchoscopy.^[15]^ The HFJV through a suction catheter distal to intratracheal tumor was applied to flexible bronchoscopy resection.

## 2 Methods

After obtaining approval from the ethics committee of Taizhou Hospital of Zhejiang Province and written informed consent, five patients were included in this study during a span of 2 years (from January 2017 to December 2018). They were scheduled for intratracheal tumor resection via flexible bronchoscope. Three men and two women age ranged from 46 to 79 (mean ± SD:60.5 ± 10.7) year, weight ranged from 40 to 71 (mean ± SD:55.4 ± 7.6) kg. The name and location of the tumor, degree of tracheostenosis, preoperative arterial blood gas analysis (FiO_2_0.37) are shown in Table 1. The common symptoms were progressive respiratory distress and cough, and relieved by nasal oxygen therapy. The patients were diagnosed with intratracheal tumor based on their symptoms, radiographic findings and tracheoscopy.

**Table 1:**
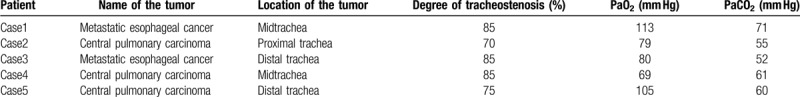
Preoperative clinical profiles.

### 2.1 Anesthetic protocol

#### 2.1.1 Preoperative preparation

All patients had fasted for 6 to 8 h prior to the procedure, and had been infused with 300 mL of ringer's solution intravenously at least. They were premedicated with ranitidine 150 mg intravenously 30 min before induction of anaesthesia.^[16]^ To prevent airway spasm, two puffs of inhaled salbutamol (0.2 mg) and intravenous methylprednisolone (80 mg) were administered after entering the operating room.

#### 2.1.2 General anesthesia

Standard monitors were applied, and invasive arterial blood pressure was monitored. Patients were preoxygenated with high flow of oxygen via face mask for 5 min before anesthetic induction. Anesthesia was induced in a step-wise manner. A bolus of dexmedetomidine (0.6 μg/kg) was administered within 10 min, and then 0.6 μg·kg^−1^·h^−1^ of dexmedetomidine as a continuous background was infused. When a loading dose of dexmedetomidine infusion was completed, ketamine was pumped at 80 mg·h^−1^ after a bolus of 30 mg. Propofol was infused at a plasma target concentration of 1.5 μg/mL initially. Then, the plasma target concentration was adjusted to maintain bispectral index values between 40 and 60. No muscle relaxant was used during the whole procedure.

#### 2.1.3 Superior laryngeal nerve block

After loss of consciousness, all the patients received ultrasound-guided Superior laryngeal nerve (SLN) block bilaterally with 1% lidocaine, 2.5 mL on each side. The linear 6 to 13 MHz ultrasound probe was placed over the submandibular area with parasagittal orientation. By the out-of-plane approach, lidocaine was injected between greater horn of hyoid and thyroid cartilage just superficial to the thyrohyoid membrane.^[17,18]^

#### 2.1.4 Topical anesthesia, suction catheter intubation and HFJV

**Figure 1:**
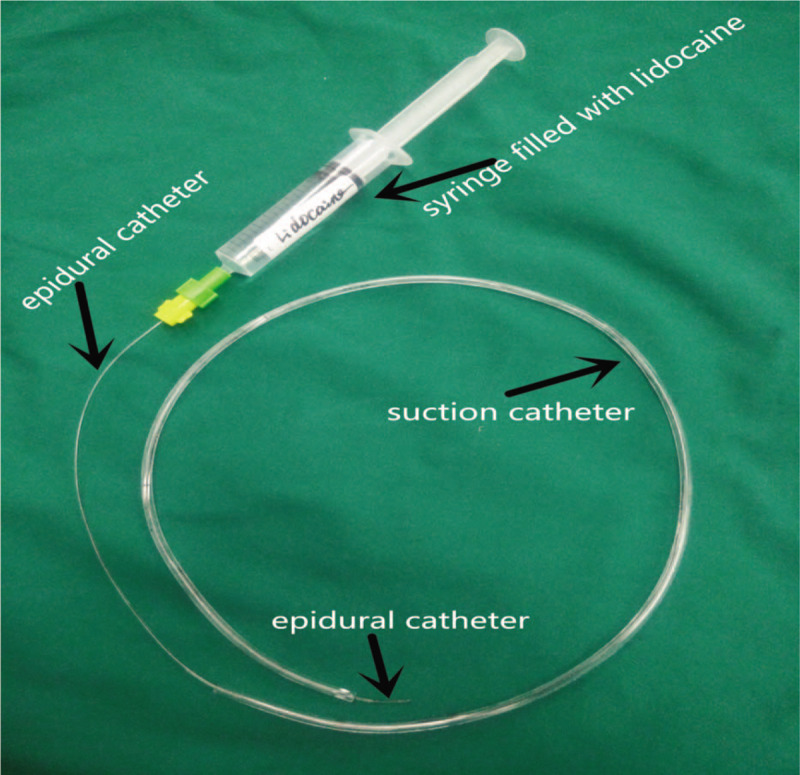
The epidural catheter inserts through the suction catheter.

**Figure 2:**
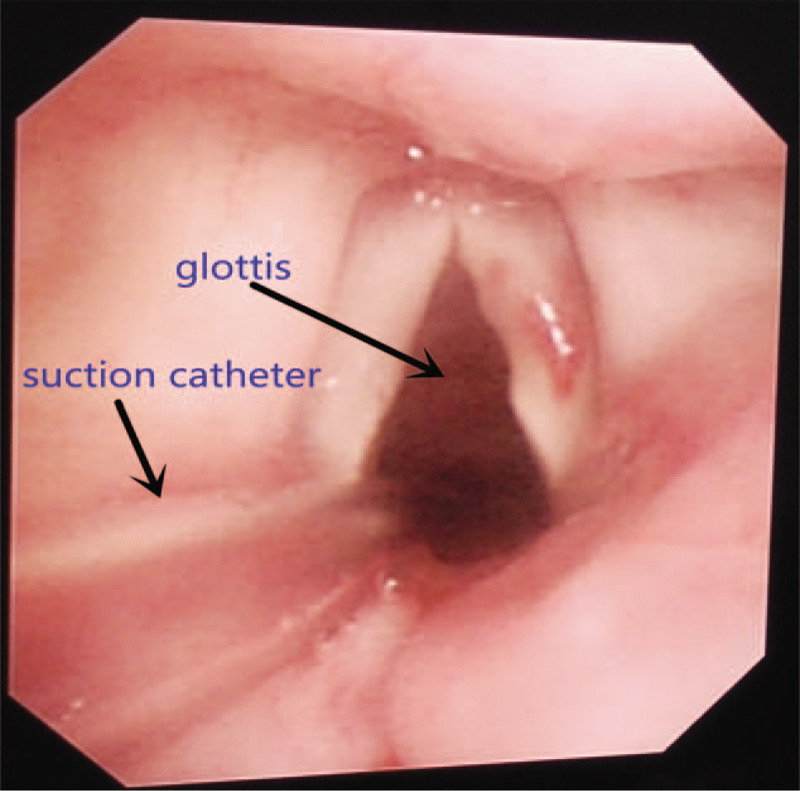
The suction catheter passes through the glottis.

**Figure 3:**
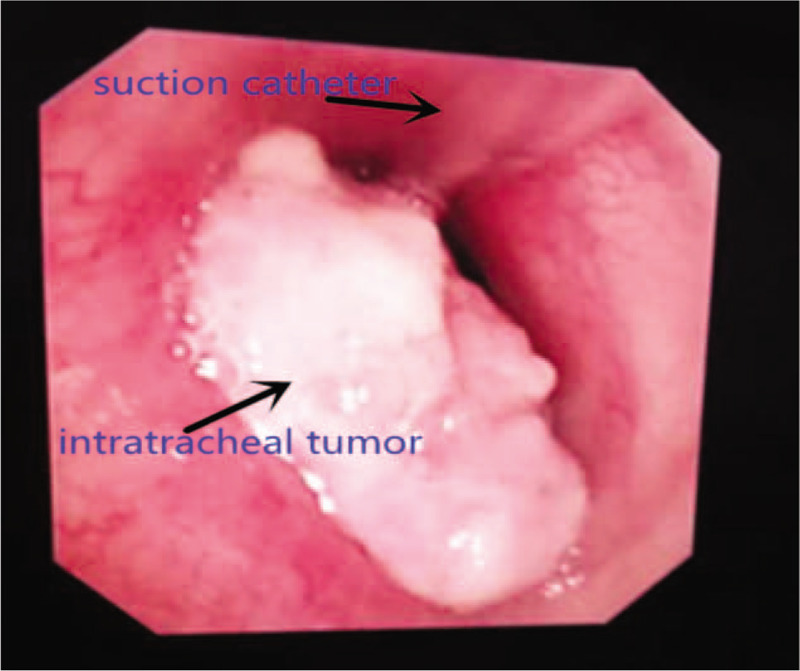
The suction catheter passes through the tracheostenosis.

**Figure 4:**
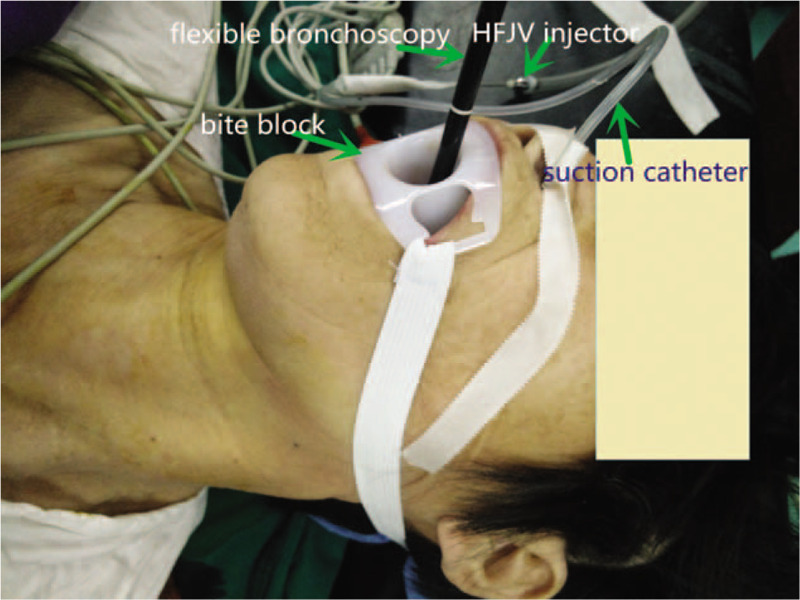
HFJV injector is connected to the suction catheter.

Laryngopharynx topical anesthesia was achieved by spraying the pharynx and larynx with 5 mL of 1% lidocaine. The connector of a 10 Fr endobronchial suction catheter (Shiley^TM^, OD 3.33 mm) was cut away. An epidural catheter lubricated with paraffin oil was inserted through the suction catheter with 2 cm beyond the tip (Fig. 1). The catheter was introduced transnasally into the trachea under direct vision with the aid of the laryngoscope and McGill forceps (Fig. 2). An additional 10 mL of 1% lidocaine was sprayed into the trachea in a spray-as-you-go manner via the epidural catheter. The catheter then passed through the tracheostenosis with the tip of the suction catheter proximal to the carina under direct vision with the aid of fiber bronchoscope. The catheter can be positioned in either of the two mainstem bronchi during the procedure if the tumor is close to the carina. The suction catheter was taped at the nose after the epidural catheter was withdrawn from it (Fig. 3). HFJV was then performed through the suction catheter with FiO_2_1.0 (Fig. 4).

The adequacy of ventilation was initially assessed by observing the movement of chest wall. The desired jet ventilation settings were then controlled by adjusting the driving pressure (usually 0.15–0.20 MPa), inspiratory/expiratory time ratio (I/E 1:2–1:3), and respiratory rate (60–100 cycles/min).^[19]^ Ventilatory parameters were monitored with pulse oximetry and arterial blood gas analysis.

The front part of bronchoscope was lubricated with compound lidocaine cream (1:1 eutectic mixture of lidocaine 2.5% and prilocaine 2.5%).^[20]^

#### 2.1.5 Posture of patients and other operation details

The patients were placed in a 30° Trendelenburg position in order to prevent hemorrhage and secretions from flowing into distal airway. The tumor of the narrowest tracheal lumen was resected first, which is beneficial to improvement of carbon dioxide expiration.

## 3 Results

The duration of surgery ranged from 90 to 125 (mean ± SD:114.8 ± 12.5) min. All the procedures proceeded uneventfully. Patients remained hemodynamically relatively stable during the whole procedure. The S_P_O_2_ was maintained above 97%. Carbon dioxide retention was alleviated obviously, when adequate patency of the trachea lumen was achieved about 30 min after the beginning of the surgery. The intraoperative arterial blood gas analysis shown in Table 2. Body movement and patient coughing/bucking were not observed. There were no common adverse events including airway spasm, massive hemorrhage, arrhythmias, pneumothorax and airway perforation. HFJV was ceased and all patients had satisfactory spontaneous breathing at the end of the procedures.

**Table 2:**
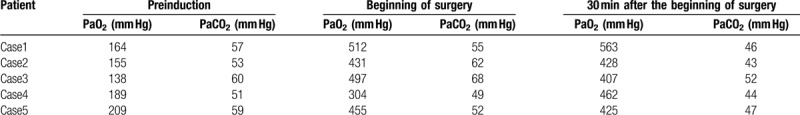
Intraoperative arterial blood gas analysis.

## 4 Discussion

Resection of a large tracheal tumor with severe tracheostenosis via flexible bronchoscope remains a formidable challenge to anesthesiologists. It is usually regarded as a palliative treatment for severe airway obstruction caused by advanced, inoperable malignant tracheal tumors. Airway obstruction is easily and critically compromised during the procedure. Suffocate is a leading cause of death. Any inappropriate manipulation of the airway will further increase oxygen consumption leading to hypoxia and probable cardiac arrest.^[21]^

The LMA and ETT are common artificial airways for treatment tracheostenosis by flexible bronchoscopy intervention.^[3,4,15]^ As for severe trachea obstruction which is an obliteration of at least 70% of tracheal lumen, LMA and ETT may not provide sufficient ventilation because of distal severe obstruction.^[13,14]^ The jet catheter of HFJV positioned proximal to the tracheostenosis may also encounter this risk. Therefore, the jet catheter was inserted distal to tracheostenosis in our cases, which ensured the good supply of oxygen. Compared with proximal airway ventilation, another advantage of placing the jet catheter distal to tracheostenosis during the surgery is that the airflow can blow the haemorrhage and tissue fragments of tumor outwards. However, previous studies have not emphasized the importance of the catheter position. Hautmann et al reported a technique of HFJV using a 14F nylon catheter placed in the trachea, either proximal to or passing through the lesion, for stent implantation or endobronchial balloon dilation in endobronchial stenoses with the flexible fiberscope.^[15]^

The endobronchial suction catheter (Shiley^TM^) is not easy to be compressed, which is stiffer than other suction catheters, and therefore the ventilation is not interrupted during the procedure. A bronchial blocker of Univent can be used for HFJV during carinal resection.^[22]^ However, the use of the endobronchial suction catheter for HFJV is simpler and more economical for bronchoscopy intervention.

In addition, placing patients in a 30° Trendelenburg position has an effect of drainage to avoid distal airway blocking.

At the beginning of the surgery, carbon dioxide retention was relieved gradually along with the tumor resection. To resect the tumor located in the narrowest lumen first and adjust the I/E to increasing expiratory time are beneficial for discharging CO_2_ and decreasing the incidence of pneumothorax. Acute moderate CO_2_ retention (60–100 mm Hg) is not associated with serious consequences among patients, thus ensuring oxygen supply is a priority.^[23]^

Our cases were of severe tracheal obstruction about 70% to 85% cross-sectional area of the tracheal lumen. It would be disastrous if an airway spasm occurred. Methylprednisolone and salbutamol were administered as routine. Preoperative treatment with methylprednisolone and salbutamol for patients with airway hyperreactivity can minimize intubation-evoked bronchoconstriction effectively.^[24–26]^

Ketamine has sedative and analgesic properties without causing respiratory and circulatory depression, and it is still a potent bronchodilator. Subanesthetic doses of ketamine have been shown to provide analgesic effect. Some studies encourage using ketamine combined with propofol for adult sedation during flexible bronchoscopy.^[27,28]^ Dexmedetomidine is a selective α-2 adrenergic agonist with several desirable pharmacologic properties, including the effects of sedation, analgesia, amnesia, antisialagogue effect, and a unique respiratory-sparing effect.^[29,30]^ A low dose of ketamine and dexmedetomidine combined with propofol target-controlled infusion was a reasonable option for hemodynamic and respiratory stability in our cases. No muscle relaxant was given throughout the procedure in view of a possible airway collapse following neuromuscular blockade.^[31]^

Superior laryngeal nerve block, a valuable technique for provision of upper airway anesthesia,^[17]^ has been applied to decrease the perioperative stress response of endoscopic laryngosurgeries.^[32]^ Ultrasound guided SLN block is more successful than blind method. SLN block combined with laryngopharynx and tracheal topical anesthesia reduces the dosage of sedative and analgesic. Furthermore, topical anesthesia must not be performed awake to avoid a potential life-threatening coughing fit.

The selection of appropriate ventilation method is fundamental. Although cardiopulmonary bypass or extracorporeal membrane oxygenation is attractive for this procedure, increase the risk of lung hemorrhage and bleeding from the tumor, with subsequent risk of impaired lung function.^[33]^ However, if the obstruction is above 85% of the trachea, we still recommend the cardiopulmonary bypass team should be on stand-by as a last resort in case that significant cardiorespiratory decompensation is to occur.

In conclusion, HFJV at the distal end of tracheostenosis is a suitable ventilation strategy during flexible bronchoscopic resection of a large tracheal tumor. However, the number of cases is small, so the method needs further study.

## Author contributions

**Conceptualization:** Guangqiu Zhu.

**Investigation:** Guangqiu Zhu, Xiaomai Wu, Donghang Cao.

**Methodology:** Guangqiu Zhu, Xiaomai Wu.

**Supervision:** Guangqiu Zhu.

**Writing – original draft:** Guangqiu Zhu, Xiaomai Wu.

**Writing – review & editing:** Guangqiu Zhu, Xiaomai Wu, Donghang Cao.
